# Social determinants of health and health inequities in Nakuru (Kenya)

**DOI:** 10.1186/1475-9276-8-16

**Published:** 2009-05-14

**Authors:** Esther Muchukuri, Francis R Grenier

**Affiliations:** 1 Community Health Worker, Eldoret, Kenya; 2 WHO Centre for Health Development, Kobe, Japan

## Abstract

**Background:**

Dramatic inequalities dominate global health today. The rapid urban growth sustained by Kenya in the last decades has created many difficulties that also led to worsening inequalities in health care. The continuous decline in its Human Development Index since the 1990s highlights the hardship that continues to worsen in the country, against the general trend of Sub-Saharan Africa. This paper examines the health status of residents in a major urban centre in Kenya and reviews the effects of selected social determinants on local health.

**Methods:**

Through field surveys, focus group discussions and a literature review, this study canvasses past and current initiatives and recommends priority actions.

**Results:**

Areas identified which unevenly affect the health of the most vulnerable segments of the population were: water supply, sanitation, solid waste management, food environments, housing, the organization of health care services and transportation.

**Conclusion:**

The use of a participatory method proved to be a useful approach that could benefit other urban centres in their analysis of social determinants of health.

## Background

Dramatic inequalities dominate global health today, where the conditions in which people grow, live, work and age have a powerful influence on their health [1]. Evidence suggests that this is particularly true in cities- where half of the world's population currently live- and that the urban setting is a social determinant of health in itself [2]. Indeed, living in cities increases exposure to unhealthy environments, disasters, climate change, violence and injuries, tobacco and other drugs, and epidemics [2].

Kenya has been particularly shaken by the changes brought by its rapid urban growth, estimated at 6% annually [3]. The country has seen slow but continuous decrease in its Human Development Index since the beginning of the 1990s, in contrast even to the slowly rising average of Sub-Saharan Africa as a whole. Very large gaps in well-being and life chances continue to exist in Kenya, which is 148th out of 177 countries ranked [4].

The Kenyan Ministry of Health has implemented initiatives to promote people's health since the independence of the country in 1963, which allowed for a substantial decline in infant and child mortality until the early 1990's [5]. Most of these programmes have however been disease-oriented, and there have been very few strategies in the past targeting social and environmental factors, particularly into assessing their effects on the health of the population at a local level.

While it is said that everyone should be on the pathway to health, there are many factors that can cause a person to start heading towards disease [6]. Notably, poverty and economic status play a very important role in dictating opportunities in employment and working conditions, educational level and access to quality health care.

Integrated interventions that support community action through participation and empowerment have been shown to reduce health risks, improve health outcomes, and promote better quality of life [2].

As part of WHO Kobe Centre's Healthy Urbanization Project, "Optimizing the Impact of Social Determinants of Health on Exposed Populations in Urban Settings", and in collaboration with the WHO Regional Office for Africa and the WHO Country Office for Kenya, it was agreed that a major Kenyan city should be included within the project and a situational analysis commissioned.

Nakuru, the fourth largest town in Kenya with a population of approximately 300 000 inhabitants [7], was chosen for this situational analysis for various reasons. Despite a life expectancy that is higher than the national average [8,9], the city is still facing many health challenges. Several difficulties have been the result of its tremendous growth associated with its status as a major administrative and commercial centre. An annual rate of population growth of approximately 7% [3] over the past three decades–compared to a national rate of 2.6% [10]–has led to a dramatic increase in demand for basic services and infrastructure, a challenge for the municipal authorities. Growth in the Sudanese refugee population is another stressor due to the poor health and immunization status of newcomers (Table 1).

**Table 1:** Demographic, economic and health indicators in Kenya and Nakuru

	Kenya	*unit*	Nakuru
Population	35.1 (2006)^a^	Millions	276 263 (2005)^d^
Population growth rate	2.6 (2006)^a^	%, annual	7 (2007)^e^
Density of population	49.0 (1999)^b^	Persons per km^2^	5 402 (2007)^d^
Infant mortality	78.0 (2005)^c^	Per thousand	42.3 (1999)^f^
Total fertility rate	5.0 (2005)^c^	Per woman	4.9 (1999)^f^
Life expectancy at birth	51.0 (2005)^c^	Years	55.6 (1999)^f^
Physicians	0.14 (2002)^c^	Per thousand	n/a

^a^[8], ^b^[9], ^c^[5], ^d ^[7], ^e ^[11], ^f^[10]

### Current initiatives in Nakuru

In 2000, Nakuru became the first town in Kenya to produce a Strategic Structure Plan, which included a citywide analysis of short and long-term solutions to its existing urban challenges. Key strategic areas were selected as part of an Intended Spatial Structure (ISS) with an overall goal of guiding Nakuru's expansion and achieving sustainable development until the year 2020. Three pillars form the structure of this plan:

1. Vision: Working towards a long-term vision and a desirable spatial structure.

2. Action: Formulating and implementing action plans able to remove obstacles for development;

3. Communication: promoting interaction and participation of stakeholders and enhancing the resolution of disputes.

Nakuru was also the first town in Kenya to implement in 2002 "Local Agenda 21", an initiative created and supported by UN-HABITAT and the Government of Belgium. The introduction of various action plans under this scheme has permitted the revitalization of the public housing stock, a rationalization of space in public areas, environmental planning of geologically-sensitive areas of the town, improved solid waste management and an optimization of municipal budgeting.

## Methods

This analysis is the fruit of field surveys conducted of the varied urban settlements; four focus groups were convened in March 2007 with political and religious figures, healthcare personnel, representatives from non-governmental organizations and community members; and interviews with key government officers (Table 2). A thorough literature review performed in 2007 and 2008 provided further qualitative and quantitative data.

**Table 2:** Participants to the focused group discussions

1. Mayor and vice-mayors
2. Religious leaders
3. Public health officer(s)/technician(s)
4. Health facility managers
5. Village elders
6. Local councilors
7. NGO representatives
8. CBO leaders
9. Persons Living with HIV/AIDS
10. Home-based care providers
11. Village health workers
12. Traditional birth attendants

A framework provided by WHO Kobe Centre was utilized to initiate discussions between stakeholders on the urban situation. From these proceedings, several social determinants of health were abstracted as causing health inequity in this particular setting. The methodology was reviewed internally by the programme officers.

We also identified strategic directions and actions that need to be considered in addressing the health inequities faced by vulnerable populations in the city, taking into consideration the role of the individual, the whole community, health systems, city management and the state. In addition, we reviewed current and past activities that act on social determinants of health and made sector-wide recommendations on how to integrate health promotion with the work of all public policy players in the city. Areas identified to affect unevenly the health of the most vulnerable segments of the population were: water supply, sanitation, solid waste management, food environments, housing, the organization of health care services and transportation.

## Results

### Water supply

The present water demand for Nakuru is estimated to be 100 000 m3/day, while water supply can currently only provide half of this amount. This shortage, particularly severe in the densely settled, low-income areas of the city, is seriously aggravated by the failure of the water distribution network to expand in line with the increase in population. The WHO report Water for Life [11] has described the consequences for poor people without adequate access to water: diarrhoeal diseases, worm infections and other infectious diseases spread via contaminated water, and lack of water makes it difficult for families to maintain basic hygiene around the home.

For those without access to the water distribution network, water contamination occurs commonly during drawing, transportation and storage, causing water borne diseases such as typhoid, cholera and dysentery, and water-based diseases such as schistosomiasis and onchocerciasis. The incidence of diarrhoeal infections is in fact significant within the total burden of disease in the city [12] (see: Table 3), with indeed an increased prevalence in those neighbourhoods without access.

**Table 3:** Most common health conditions in 5 municipal health centres of Nakuru (2006)

1. Upper Respiratory Tract Infections
2. Malaria
3. Gastroenteritis
4. Dermatitis
5. Broncho-pneumonia
6. Traumatic wounds
7. Bronchitis
8. Gastritis
9. Abscesses
10. Typhoid fever

Source: Nakuru Public Health Department

Beside the inadequate supply, the high cost of maintenance of an obsolete distribution system and the lack of a clear policy on private and public participation and the associated costs are problematic. The shortage and deficient governance have also given rise to a black market in water, with its intrinsic health and social risks.

Previous research suggested that in less wealthy regions, the benefit of a $1 investment in improved water supply has a value ranging from $5 to $28, as clean water affords people healthier bodies, better food, more sanitary households and bodies, stronger and livelier children, and more productivity in work [13].

For Nakuru, we identified several recommendations that should be implemented to improve water supply and water quality, including minimizing pipe leakages through weekly inspections; maintaining a consistent distribution pipe diameter to reduce surge bursts; improving account receivables management; formulating clear policies to guide and regulate water distribution; and extending the distribution system to cover low-income zones.

The Nakuru Water and Sanitation Services Company Limited, The National Water Conservation and Pipeline Corporation, the Kenyan Army, eight private boreholes providers and other private organizations that manage water supply sources and the central government representatives for the area were key stakeholders identified.

The Nakuru Environmental Management Project (NEMP), aimed at improving environmental management capacity within the city, was initiated in 2005 under the direction of a Joint Coordinating Committee from the local government and implemented by the management companies of the supply sources. A target of four years has been set to significantly improve water quality monitoring, environmental management, coordination in watershed management and increased public and private participation. Other key stakeholders include the national park administration, the wildlife service and the water testing laboratory.

### Water sanitation

In Nakuru, only 19% of the habited areas have access to the sewerage reticulation system, a lesser proportion than those with potable water. Many residents rely on cesspools and septic tanks, available mainly in high-income areas, while the majority either have access to public latrines or dispose of their waste openly and indiscriminately. Again, the poorest residents living in densely populated areas are those with the least infrastructures available to them.

This situation has serious health consequences and is the main cause of transmission of faecal-oral (typhoid, dysentery, cholera) and water-related vector diseases (malaria, yellow fever, sleeping sickness) in the community. Some children even play in the stagnant sewage, putting their health at great risk.

Other issues of importance that affect wastewater management are the frequent blockages due to poor maintenance, ineffective surface water drainage that causes frequent overloading and inefficient revenue collection–less than 45% of the amount due–and air pollution caused by the foul odour of the surface, stagnant raw sewage.

The Nakuru Water and Sanitation Services Company Limited and the Nakuru Environmental Consortium are the major players in charge of wastewater management. On this issue, town elected officials and the Constituency Development Fund are also important stakeholders.

Recommendations and targets that have been identified include increasing sewage coverage to a minimum of 80% of dwellings, particularly improving access in low-income zones; matching the provision of potable water to that of sewerage; conducting weekly inspections to eliminate frequent blockages; treating the toxic waste before it enters the municipal sewage system; and providing drainage for surface run-off.

### Solid waste management

The management of solid wastes in Nakuru town is primarily under municipal jurisdiction and responsibility. However, in recent years, private sector entrepreneurs have increasingly been involved in refuse collection and disposal. Municipal collection services cover most of the old town and high-income areas, but exclude some of the outlying, newly and poorest developed areas, where many rely on private initiatives.

The municipality has one open dumping site, but the majority of refuse is abandoned in undesignated sites and either burnt or left unattended. Similar to what has been happening in many urban areas, pollution caused by non bio-degradable substances, such as plastic bags, is also another major concern.

Inadequate disposal techniques accentuate the ability of animal vectors to propagate infectious diseases. It also attracts scavengers, which results in the creation of a public nuisance. The heaps also contaminate ground, air and surface waters.

Proper waste management involves planning, forecasting, organization and execution of the various aspects of solid waste: generation, collection, transportation and disposal. In Nakuru, there are several issues that hinder correct waste management, such as inadequate funding and poor revenue collection, inadequate equipment and personnel, low public awareness on environmental health, poor location and management of the existing refuse disposal site and finally, indiscriminate disposal of solid waste-particularly non-biodegradable material-by the residents.

Several actions should be taken by the stakeholders, which are the municipality, environmental NGOs and community-based organizations. We identified establishing weekly waste collection routes, increasing funding and staffing of waste-related activities and implementing awareness campaigns as the most important actions to improve solid waste management.

### Food environment

In the context of the alarming rate of HIV, immunosuppressed patients have been singled out as persons requiring higher levels of nutrients that can boost their immunity. Nakuru has one of the highest HIV prevalence rates in the country, reported to be 24.6 percent [8]. Under the Comprehensive Care Programme initiated by the central government, Rift Valley Provincial Hospital patients have been supplied with targeted food supplements; i.e. children borne of HIV-positive mothers are now supplied with breast-milk and vitamin A supplements.

The government, in cooperation with private organizations, has also implemented the School Feeding Programme, an initiative that has helped children in many marginalized areas where crop failure is a severe barrier to nutritional intake. Vitamin A has also been given as part of the national vaccination programme to boost proper development. Maternal and child health clinics in municipal health centres focus on giving appropriate nutritional advice to mothers and monitor growth. These approaches target the early detection of children who are potentially at risk of malnutrition and initiate corrective measures at an early stage.

Nevertheless, several problems remain regarding food hygiene and inadequate nutritional intake. Health issues caused by malnutrition–as a result of lack of food or ignorance of required daily allowances–and food poisoning arise. Individual preferences, lifestyles and culture also affect dietary habits; on the other end of the scale, there is an increase in prevalence of both obesity in high-income households, and obesity-related illnesses, i.e. high blood pressure and diabetes.

Recently, the undernutrition of the elderly has been under the spotlight because of a growing trend toward nuclear families. In these circumstances, many elderly people find it difficult to shop for groceries and are unable to cook their own meal because of their frailty, leading to unbalanced diets and inadequate nutritional intake.

Food environments are relevant social factors that affect diets. It is important to examine social factors related to individuals' eating habits and continue research efforts to improve food environments. It is envisioned that such efforts will contribute to the success of public health promotion policies and programs implemented by the Kenyan government.

Challenges faced within food environments include the mushrooming of informal, unplanned and unaccredited food stands that are a significant health threat, especially because they often target young people near schools.

The municipality, the ministries of agriculture and health and the community-based organizations and NGOs dealing with the nutrition of street children must collaborate on this particular issue. Curtailing the development of unplanned and unsanitary food outlets is one approach that is advocated, along with appropriate and enhanced law enforcement.

### Housing

Planning for housing needs to parallel the rapid growth of the urban population and its well-being. Suitable lands need to be available and appropriate and sufficient number of dwellings built to support economic development. It is estimated that the majority (87%) of Nakuru residents are tenants and that a significant 13% own their homes.

There are at least 6956 public housing units within Nakuru town, owned by the municipality or the central government and available to middle to low income residents. Municipal houses have good amenities and are close to social halls and health facilities. However, there have been very few new public housing units built recently in spite of the growing need. Public housing has also been subject to poor maintenance and deterioration.

The private sector is the largest provider of housing in the town and the rate of expansion has been rising rapidly. Private housing for those on high and middle incomes offers access to potable water, sewerage installations and septic tanks. Private, informal housing occurs in several areas and is faced by a number of problems including poor planning, inadequate support infrastructure such as roads, drainage, garbage collection, water, electricity and inadequate public spaces. Overcrowding is a common occurrence in this poorly-developed setting where buildings often do not meet minimal building standards or other minimal social, aesthetic and environmental characteristics.

Informal constructions are serious health hazards because of their poor design and insanitary conditions (damp, improper lighting and ventilation) that promote injuries and the transmission of infectious diseases. Some are also built in disaster-prone areas and are a "ticking timebomb" to their residents.

Other challenges facing housing in the municipality include the lack of adequate enforcement of building standards, poorly planned expropriation of lands and houses and the difficulty for women to become owners and obtain mortgages.

As part of the proposed solutions, we have identified increasing enforcement of building regulations; the allocation of greater funds for maintenance; and construction of public housing units and initiatives to assist home ownership among women and other disadvantaged groups.

Key stakeholders include the Ministry of Housing, the municipality, local business organizations, the architectural association, tenants and other community-based associations.

### Organization of healthcare services

Both public and private health facilities operate within the city borders, with NGOs and herbalists completing the offer of services.

The Ministry of Health operates the main hospital, that has a capacity of 715 beds and 46 cots; it serves 1.5 million people over 7291 square km and handles an average of 27 000 admissions and 170 000 outpatients every year. The municipality operates a maternity ward, a health centre and four dispensaries.

In comparison, the private sector has 210 self-employed health practitioners, numerous herbal clinics, faith-based dispensaries, one maternity clinic, four major hospitals and two nursing homes. A national association also runs one family planning clinic in the city.

The public health care services are clearly inadequate and overcrowded, severely affecting those unable to afford private care. Facilities face inadequate maintenance and frequent shortages of essential drugs and vaccines, with serious health consequences. A number of facilities face administrative problems in term of ownership of land. Shortage of staff is also critical. The government has acknowledged this problem and has made its health sector policy priority to make all health services more effective, accessible and affordable [14].

In term of access to emergency care, Nakuru has an inadequate first respondent system, and would benefit from having at least one working ambulance to better fulfil the needs of its residents.

Lawmakers should revise and enforce regulation of private health care facilities and required medical training. Private clinics are often staffed by unqualified personnel and alternate facilities–such as herbal clinics–cause confusion in the sector. Finally, the lack of a proper coordinating committee for all health service providers has led to surveillance problems that must be overcome. As shown in Table 4, focus groups have highlighted the need to include communities and grass-roots representatives at all the levels of planning.

**Table 4:** Key activities needed to improve the social determinants of health in Nakuru

Short-Term	Long-Term
1. Improve environmental health within the specific SDH.	1. Develop programs to reduce poverty.
2. Ensure coordination of activities carried out by all the stakeholders and mobilize intersectoral action.	2. Enhance and increase public infrastructures to match demand.
3. Boost cooperation, foster partnerships and capacity building between stakeholders.	3. Develop a strategic health plan that will involve all stakeholders, including children, women, elderly and other disadvantaged groups.
4. Increase health awareness and healthy lifestyle among residents through health education, promotion and advocacy.	4. Make sure that public policies are compatible with healthy living and urbanization.
5. Improve community mobilization and participation in health oriented programs and forum.	5. Insure good management and accountability of public funds.
6. Encourage efficiency in law enforcement, especially in the Public Health and Environment Departments of the municipality.	6. Develop practical methodologies for monitoring social gaps and inequalities quantitatively and qualitatively.
	7. Monitor and evaluate the performance of programs.
	8. Update health personnel knowledge on new technologies.

Key priorities include improving the maintenance of facilities; supporting the decentralization of services (community-base care) by increasing the number of facilities in low-income, densely populated areas; improving access and availability of essential drugs and vaccines; hiring more staff to curb shortfalls; and improving networking among all stakeholders.

### Transportation

The town is well integrated within the international, national and regional transportation systems. The Trans-African Highway (A104), which links the city of Mombasa in Southern Kenya to Kampala (Uganda), and the main Ugandan railways provide access to freight and passenger services. The town is also connected to its hinterland through national roads.

At the city-level, the municipality is responsible for 236 km of roads laid out in a grid pattern throughout the city. About 74 km are in fair condition but the rest is either poor or unusable because of the cost to the municipality of properly maintaining its road network.

Due to population and economic growth in recent years, traffic flow in the town has increased tremendously and is very evident during rush hour, when traffic police are deployed to control the flow of vehicles.

Several means of transportation are also available for public transport, with 2 500 matatus (10 to 14-person carriers), 100 minibuses, 200 buses, 50 tuk-tuks (motorized 3-wheeled light vehicles) and 3 000 bicycle taxis (boda-bodas).

Transport-related injuries are a leading cause of morbidity. With the recent advent of bicycle carriers for commercial purposes, the number of accidents has also increased dramatically. Injuries involving boda-bodas average 20 per week: due to the non-existence of demarcated areas for such carriers and other non-motorized traffic, they are forced to squeeze and compete on the main roads with other vehicles; there is also an issue with their visibility at night. With the Public Transport Reforms implemented in 2003, the number of accidents caused by matatus and buses have been reduced by over 50%.

Other problems include air and sound pollution, road blockage because of non-channeling of local and through traffic, congested interchanges and terminus, poor organization and control of non-motorized transport businesses, still-insufficient law enforcement efforts and corruption within the enforcement body.

We propose that the key stakeholders–the Ministries of Transport and Public Works, the Kenya Roads Board, NGOs and community-based organizations–introduce initiatives including segregating local and through traffic with bypasses; ensuring the integration of cyclists on the roads; and increasing the enforcement of traffic laws and regulations to curb the safety risks for boda-boda.

## Discussion and conclusion

The social determinants of health identified and considered to be relevant for reducing the health inequalities in Nakuru include the insufficient availability of water; poor waste management; changing and unhealthy food environments; deficient growth in housing supply; and the inadequate organization of transport and health care services. There are many others, such as human-wildlife conflicts in the areas that border the national park, roaming dogs and unemployment.

Provision of potable water for domestic use is felt to be the first priority for Nakuru's residents and will only be possible with an extensive rehabilitation and expansion of the existing distribution system to include lower-income areas and provision of safe water using mobile units in the meantime.

The availability of new housing has not followed the demographic expansions of the population, and should be adequately planned. Many elderly, disabled and low-income persons are unable to construct their own houses and rent relatively affordable housing. Housing environments also affect the lifestyle and health of residents.

Issues that relate to liquid and solid waste management are serious challenges to the municipality and all the agencies charged with their removal. The planning and reorganization of these services must be sought with all the stakeholders.

With regard to food environment, various problems have been identified that concern all age-groups. More effective approaches that target social and environmental factors are needed to deal with health problems such as malnutrition and food-borne diseases.

Transport is not orderly and regulatory measures should be put in place for bicycles. Roads should be improved.

Several activities and measures to optimize the impact of social determinants of health and promote health in Nakuru have been identified, both for the short and long-term (Table 4).

Key stakeholders influential to these social determinants of health are various organizations, some quite difficult to monitor; for most of them, health care is also not their prevalent activity. In order to reduce the health disparities in Nakuru, these challenges must be overcome by ensuring collaboration and coordination between all stakeholders.

Intersectoral collaboration, closer connections and communications among stakeholders to work on social determinants of health through good governance are recommended. Political goodwill should be encouraged and nurtured in order to foster collaboration in health and especially health care delivery.

Social and environmental factors have been recognized as significant points of intervention in order to reduce health disparities [15,16]. Investments in health are deemed essential in order to achieve economic growth with a productive workforce. This will be achieved only if there is more equitable access to the benefits of development, as inequities have severe health consequences and pose an unacceptable threat to human well-being and security [17].

In terms of organization of the health care system, this could only materialize by involving all segments of the population in the definition of needs and planning. Current infrastructures are inadequate, and investments into more affordable and easily accessible facilities to lower-income communities should be made.

It is appropriate for all urban stakeholders involved in urban governance to seek to understand the local determinants that reflect and influence the health of their residents. The use of community-participatory research methods similar to this strategic analysis appear to be a valid approach to gain a perspective on the social determinants of health; this technique should be the first-step in defining and monitoring action in local settings.

To mainstream social determinants of health across all stakeholders in Nakuru, our analysis allowed the identification of a series of actions that need to be undertaken (see: Table 4). This initiative fulfilled one of the recommendations identified by the Commission on Social Determinants of Health in its final report [18], and acknowledging that there is a problem is a vital step to ensure follow-up action. In order to address the Commission's recommendation in its entirety, a new project to assess intra-city inequities across various policy domains will be initiated in Nakuru in the next few months and will hopefully raise even further the political support to address these inequities.

## Competing interests

The authors declare that they have no competing interests.

## Authors' contributions

EWM carried out the focus group discussions and field surveys. FRG participated in the design of the study and coordination of the project. All authors participated in the drafting of the manuscript, read and approved its final version.
